# The influence of jittering DHS cluster locations on geostatistical model-based estimates of malaria risk in Cameroon

**DOI:** 10.1016/j.parepi.2024.e00397

**Published:** 2024-12-08

**Authors:** Salomon G. Massoda Tonye, Romain Wounang, Celestin Kouambeng, Penelope Vounatsou

**Affiliations:** a Swiss Tropical and Public Health Institute, Basel, Switzerland; b University of Basel, Basel, Switzerland; c National Malaria Control Program, Yaounde, Cameroon; d National Institute of Statistics, Yaounde, Cameroon

**Keywords:** Bayesian inference, Demographic and health survey (DHS), Malaria indicator survey (MIS), Jittering, Geostatistics, Malaria interventions

## Abstract

**Background:**

In low-and-middle income countries, national representative household surveys such as the Demographic and Health Surveys (DHS) and the Malaria Indicator Surveys (MIS) are routinely carried out to assess the malaria risk and the coverage of related interventions. A two-stage sampling design was used to identify clusters and households within each cluster. To ensure confidentiality, DHS made the data available after jittering (displacement) of the geographical coordinates of the clusters, shifting their original locations within a radius of 10 km. Our study assessed the influence of jittering on the estimates of the geographical distribution of malaria risk and on the effects of malaria control interventions using data from the latest MIS in Cameroon.

**Methods:**

We generated one hundred datasets by jittering the original MIS data. For each dataset, climatic factors were extracted at the jittered locations and Bayesian geostatistical variable selection was applied to identify the most important climatic predictors and malaria intervention coverage indicators. The models were adjusted for potential confounding effects of socio-economic factors. Bayesian kriging based on the selected models was used to estimate the geographical distribution of malaria risk. The influence of jittering was analysed using results of the variable selection and the Bayesian credible intervals of the regression coefficients.

**Results:**

Geostatistical variable selection was sensitive to jittering. Among the important predictors identified in the true data, distance to water bodies and presence of forest were mostly influenced by the jittering. Altitude and vegetation index were the least affected predictors. The various sets of selected environmental factors were able to capture the main spatial patterns of the disease risk, but the jittering increased the prediction error. The parameter estimates of the effects of socio-economic factors and intervention indicators were relatively stable in the simulated data.

**Conclusion:**

In Cameroon, the malaria risk estimates obtained from the jittered data were comparable to the ones generated using the true locations; however, jittering modified our interpretation of the relationship between environmental predictors and malaria transmission.

## 1 Background

During the last decade, Bayesian geostatistical models have increasingly been used to determine spatio-temporal patterns of malaria risk, capture the effects of control interventions, and identify environmental and socioeconomic factors that are related to changes in the distribution of malaria risk (Gosoniu et al., 2012; Adigun et al., 2015; Diboulo et al., 2016; Ssempiira et al., 2017). In most low- and middle-income countries, the data used to fit geostatistical models are mainly collected by national households surveys such as the Demographic and Health Surveys (DHS) and the Malaria Indicators Survey (MIS) (Giardina et al., 2014). A two-stage sampling design was used to select survey clusters and households within clusters. The clusters included typically around 25 households per cluster and were geo-referenced according to their centroid. However, to ensure confidentiality of the health status of the enrolled individuals, the longitude and latitude of the cluster centroids were randomly jittered (displaced) from their original positions within a radius of 0 to 10 km according to the type of location (rural / urban) (Burgert et al., 2013).

Some studies have either assessed or mitigated the influence of imprecise geographical locations on model fit (Kyriakidis and Dungan, 2001; Cressie and Kornak, 2003; Gryparis et al., 2009; Li et al., 2012; Altay et al., 2023). In particular, studies on jittering DHS data have investigated the impact of spatial displacement on the estimates of the effects of distance-based covariates such as proximity to health services or areal covariates such as poverty measures defined in areas around a cluster location. These studies have been conducted in the field of HIV infection (Warren et al., 2016a; Warren et al., 2016b) and using simulated and real data to assess the potential effects of location shift on model parameter estimates. However, within the Bayesian geostatistical modelling framework, studies assessing the effects of the cluster displacement on the pixel-level predictions of disease risk such as malaria and on the estimates of the covariates, for example climatic factors or control intervention effects are rather lacking.

The fourth DHS in Cameroon was combined with the Multiple Indicators Cluster Survey (MICS) in 2011 and carried out between January and August, a period which unfortunately did not overlap with the high malaria transmission season. In the same year, the National Malaria Control Program (NMCP), the National Institute of Statistics (NIS) and other partners conducted a MIS from September to November within the high malaria transmission season on a subset of clusters previously surveyed by DHS. The geographical coordinates of the DHS clusters involved in the MIS were registered without any alteration (Enquête Démographique et de Santé et à indicateurs multiples, 2011; Minsante, 2012; Massoda Tonye et al., 2018).

Our study assessed the influence of jittering of cluster locations on geostatistical model-based malaria risk estimates at high spatial resolution and on the estimates of the control interventions effects. A large simulation study using the jittered locations was carried out based on the MIS cluster locations and the random displacement procedure of DHS. Bayesian geostatistical models were applied on the simulated data and the results were compared with the non-jittered data.

## 2 Methods

### 2.1 Country settings

Cameroon, a country in Central Africa has a population of around 24 million inhabitants with an annual population growth of 2.5 % within the territory surface of 475,650 km^2^ (BUCREP, 2011). Fifty one percent of the population lives in urban areas (INS, 2015). In 2017, the gross domestic product rate was 3.1 % and the last estimates of human development index done in 2014 was 0.518 (Cameroon, 2024; *Human Development Reports*, 2024). The country is spanned by different ecological environments with various lengths of malaria transmission, namely: the dry Sahelian in the Far North region and Sudano-Guinean in the North region (4–6 months), the highlands of Adamawa region and West (7–12 months); the equatorial forests in Centre, East and South regions; the Atlantic coastal in Littoral, South-West and part of South regions where malaria transmission is perennial (12 months) (PNLP, 2014; Programme National de Lutte contre le Paludisme-Cameroun, 2007; Gemperli et al., 2006).

### 2.2 Data

#### 2.2.1 Malaria indicator survey data

**Fig. 1:**
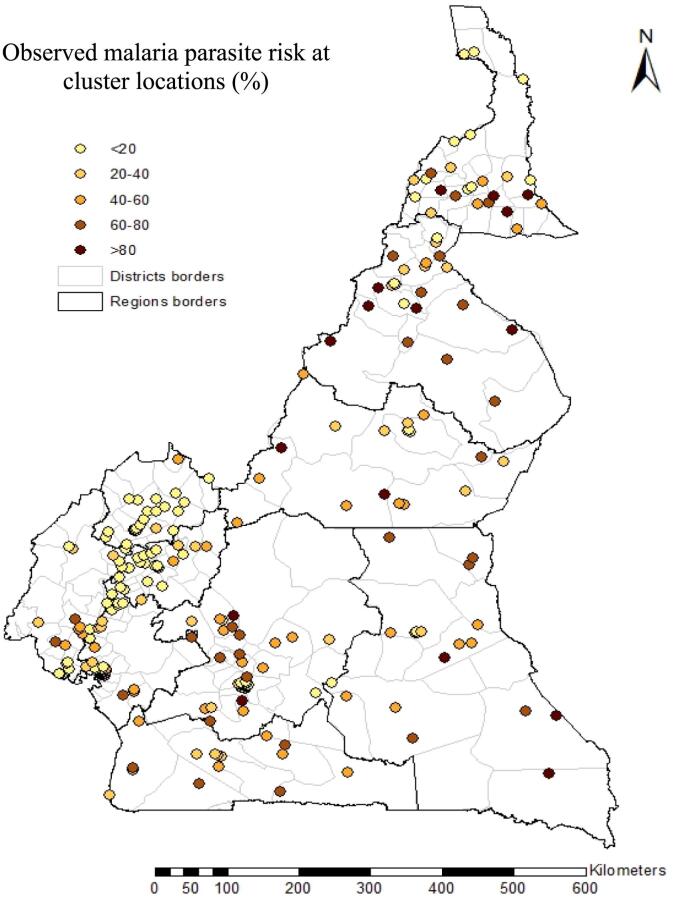
: Observed malaria parasite risk in children under 5 years at 257 MIS locations.

The Cameroon Malaria Indicator Survey (MIS) of 2011 was nationally representative and funded by the Global fund to fight AIDS, Tuberculosis and Malaria with the aim to collect malaria indicators additional to those in DHS and to compare the overall malaria parasite prevalence obtained by the MIS and DHS data (Ministry of Public Health, 2010). The MIS was conducted in 257 clusters randomly selected out of the 580 clusters of the Cameroon DHS 2011 and involved 6040 households and 4939 children aged between 6 and 59 months (Fig. 1). Rapid Diagnostic Tests (First Malaria Response Antigen) were used for malaria screening of children with the approval of adults in charge (Enquête Démographique et de Santé et à indicateurs multiples, 2011; Minsante, 2012). Apart from the malaria parasite data, the survey collected information on malaria interventions and socio-economic status proxies.

Data on malaria interventions was processed to create the following intervention coverage indicators as proposed by the Global Malaria Action Plan and Roll Back Malaria monitoring and evaluation group: (a) proportion of children in the households who slept under an insecticide treated-net (ITN) the night before the survey, (b) proportion of households in the cluster with at least one ITN, (c) proportion of households in the cluster with one ITN per two persons, (d) proportion of population with access to an ITN in their household. Adherence to the health system was calculated by the proportion of children with fever who sought treatment at hospital, tested and treated with the recommended anti-malaria drugs (Artemisinin-based combination therapy) during the last two weeks (Roll Back Malaria, 2013).

The analysis included the education level of women of reproductive age and the household welfare index as socio-economic proxies. The education level was categorized into three levels (primary, secondary and university). The household asset index was available in the database and it classified households into the poorest, poor, middle, rich and richest categories. The area type (urban or rural) was extracted from the MIS data.

#### 2.2.2 Simulated data

One hundred datasets were generated from the original MIS data, each with randomly jittered cluster locations from the MIS coordinates according to the jittering algorithm used by the DHS program. In particular, clusters in urban areas were randomly displaced within a radius of 2 km; whilst 99 % of those in rural areas were shifted within a radius of 5 km from their original locations. The remaining 1 % of rural clusters were displaced up to a radius of 10 km, as these clusters remained sparsely populated (Burgert et al., 2013). The simulated data differed from the MIS data in the cluster coordinates. The prevalence, intervention and socio-economic information were maintained the same as at the original locations.

#### 2.2.3 Environmental and climatic data

Environmental and climate proxies were obtained from satellite sources (Table A.1 in the Appendix). Day and night Land Surface Temperature (LSTD, LSTN), Normalized Difference Vegetation Index (NDVI), Enhanced Vegetation Index (EVI) and Rainfall estimates (RFE) were averaged over the year prior to the survey. The covariates forest, savannah, cropland and distance to permanent water bodies (DWB) were retrieved or calculated from Land Cover satellite maps. The data were extracted at the MIS cluster locations and at the locations of simulated datasets.

### 2.3 Bayesian geostatistical modelling

Bayesian geostatistical binomial regression models were fitted on the malaria parasite data (MIS and simulated ones) aggregated at cluster locations (See Additional file 1). The models incorporated geostatistical variable selection to identify the most important climatic and environmental covariates including their functional forms (i.e. continuous or categorical). The categorical covariates were derived by analyzing the relationship between malaria cases and continuous climatic predictors. The cut-off points were validated using linear regressions. In particular, a categorical indicator was created from each climatic predictor, taking the values 0, 1 and 2 which corresponds to the exclusion of the predictor from the model or its inclusion in continuous or categorical form, respectively (See Additional file 2). It was assumed that the indicator arose from a multinomial distribution with probabilities defining the variable-specific exclusion/inclusion probabilities (in continuous/categorical forms) in the model. A threshold of 50 % was considered for the probability of inclusion (i.e. posterior inclusion probability) into the predictive geostatistical model (Barbieri and Berger, 2004). The predictive performance of the models obtained from each simulated dataset was evaluated using the log predictive score comparing model-based predictions at the MIS locations with the observed MIS survey data (Krnjajić and Draper, 2014).

Bayesian kriging as described in the additional file 1 (Diggle et al., 2002) was separately applied on the observed data as well as on the simulated data with the best and least predictive performance (i.e. maximum and minimum log predictive score, respectively) namely Model 1a (MIS data), Model 1b (simulated data with the best predictive performance) and Model 1c (simulated data with the worst predictive performance). For each of the above models, a gridded surface of malaria parasite risk was estimated over 117,192 cells/pixels of 2 × 2 km^2^ spatial resolution covering the country.

To assess the effect of jittering on individual level covariates such as the ITN coverage indicators, geostatistical Bernoulli models were fitted on individual level data obtained from the observed MIS and the simulated data. As described above, three models were fitted i.e. Model 2a (applied to MIS data), Model 2b and 2c (applied to simulated data) with the best and worse predictive ability, respectively. We implemented geostatistical variable selection to identify the most important ITN indicators (Giardina et al., 2014). The individual level models were adjusted for the confounding effects of the climatic predictors selected by the corresponding cluster-level model and the socio-economic proxies. Due to high correlation among the ITN indicators, only one indicator was allowed into the model.

Covariates were statistically important when the corresponding Bayesian Credible Interval (BCI) did not include the one in the odds ratio scale, so the covariates were statistically significant if they did not include 0 in their BCI. Computation was performed on a dual processor workstation (2 × 2.6 Ghz, 128GB RAM). OpenBUGS version 3.2.3 (Imperial College and Medical Research Council, London, UK) was used for Bayesian model fit and prediction (Lunn et al., 2000). Data management and analysis were carried out in R statistical software (Sturtz et al., 2005; R Core Team, 2016). Convergence was assessed by the Geweke statistic, visual inspection of the traceplots and achieved in less than 200,000 iterations (Geweke, 1991). Maps were drawn in ArcGIS version 10.2.1 (http://www.esri.com/) (ESRI, 2013).

## 3 Results

### 3.1 Descriptive analysis

The overall malaria prevalence estimated by the MIS data was 33 %. In the rural areas, 43 % of children were tested positive, meanwhile this proportion was 19 % in urban areas. The most affected areas were located in the North, East and South regions of Cameroon with a malaria risk of 57.2 %, 56.5 % and 50.9 %, respectively. The proportion of mothers that had attended university was 6.7 % and those without any education were 23.3 %. The proportion of households with at least one ITN was 46 %. Only 9 % of the population had access to an ITN in their household and 12 % of children with fever who sought treatment at hospital received a recommended Artemisinin-based combination therapy (ACT) during the last two weeks. Sixty-eight percent of households were most poor or poor.

**Fig. 2:**
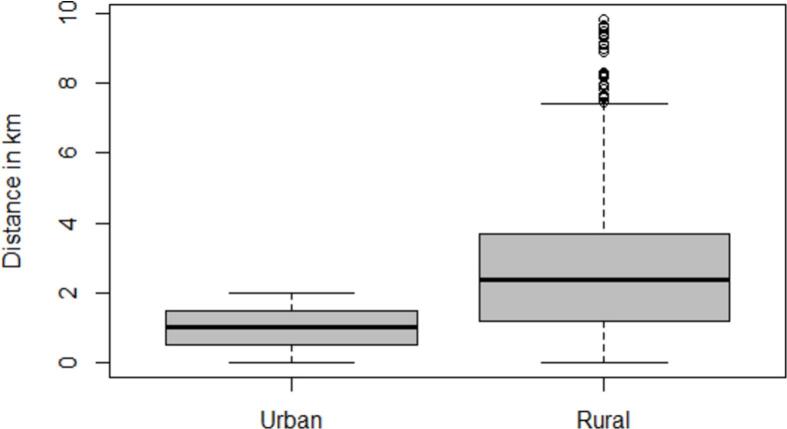
: Distribution of the distances (km) between the original and shifted locations across the 100 simulated datasets according to urban and rural cluster type.

**Fig. A.1:**
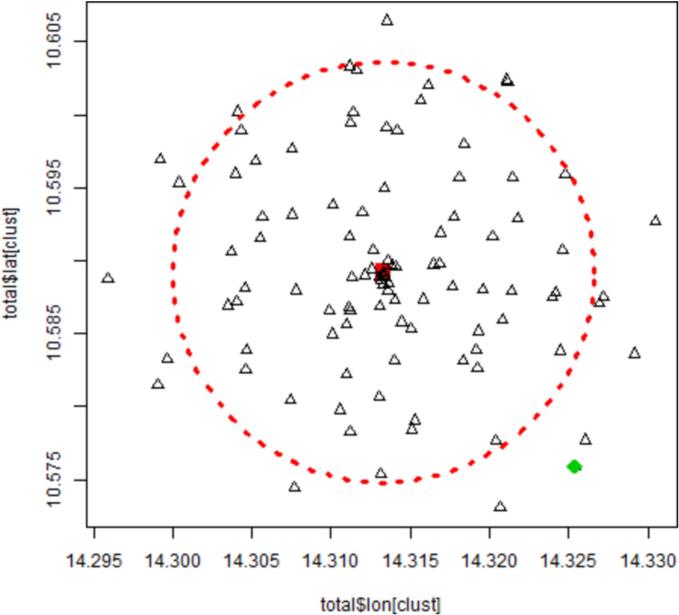
Cluster location 12 and jittered locations.

**Fig. A.2:**
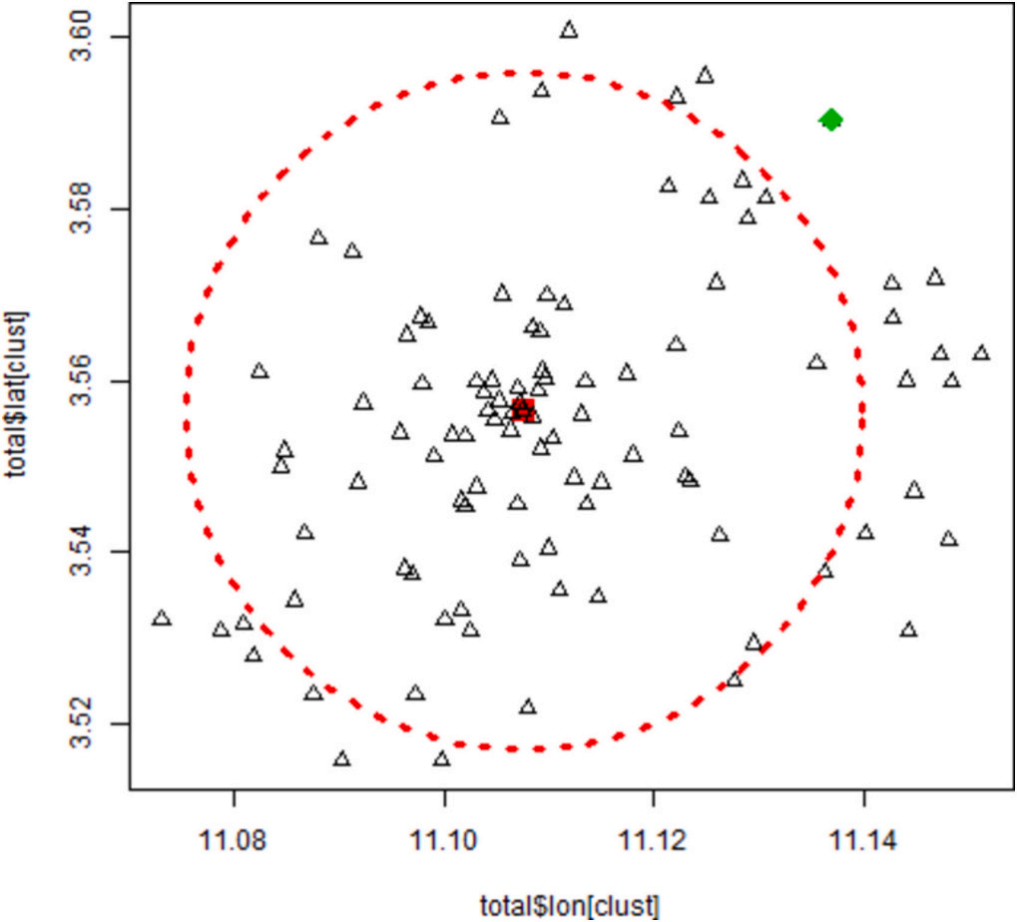
Cluster location 49 and jittered locations.

**Fig. A.3:**
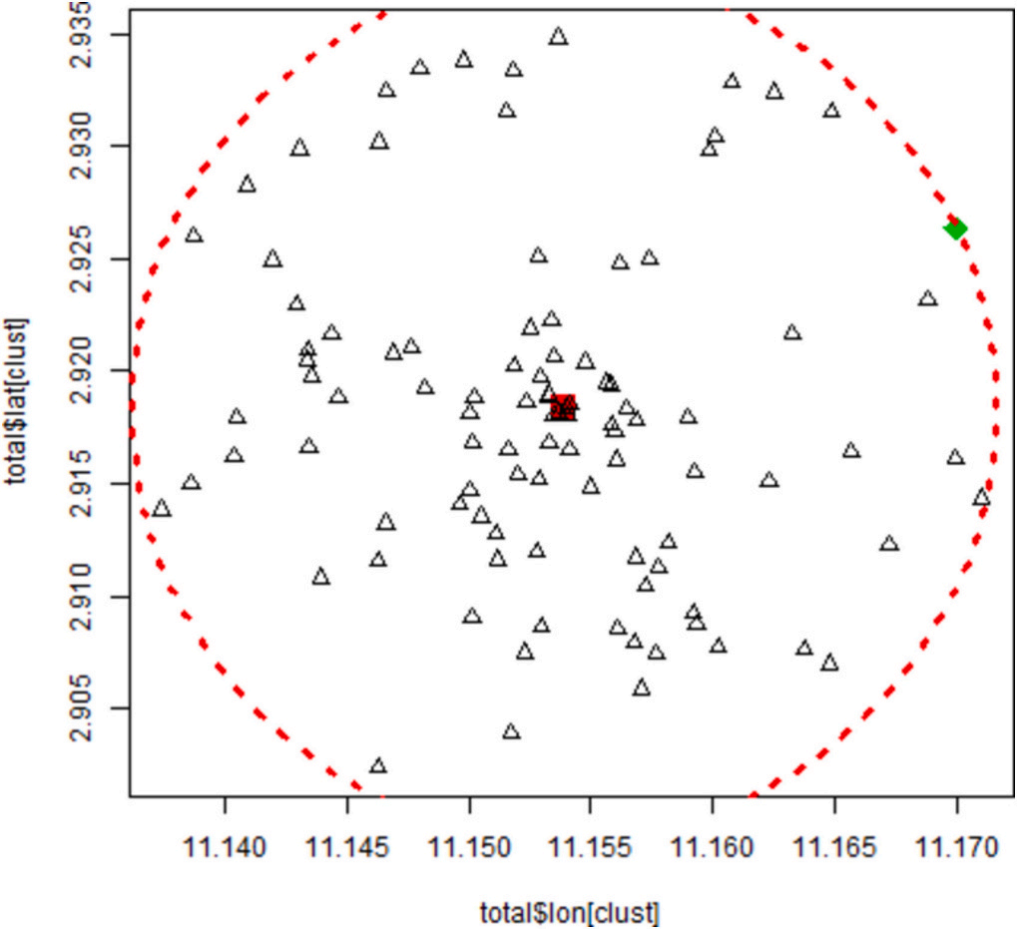
Cluster location 36 and jittered locations.

**Fig. A.4:**
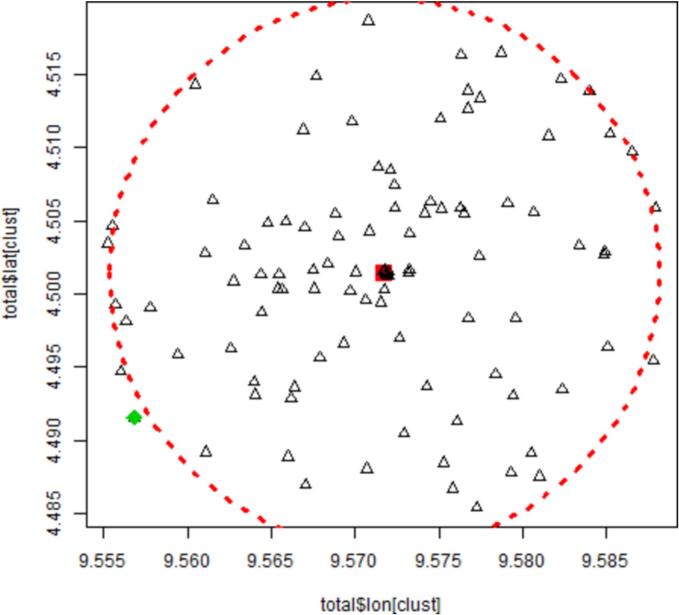
Cluster location 126 and jittered locations.

**Fig. A.5:**
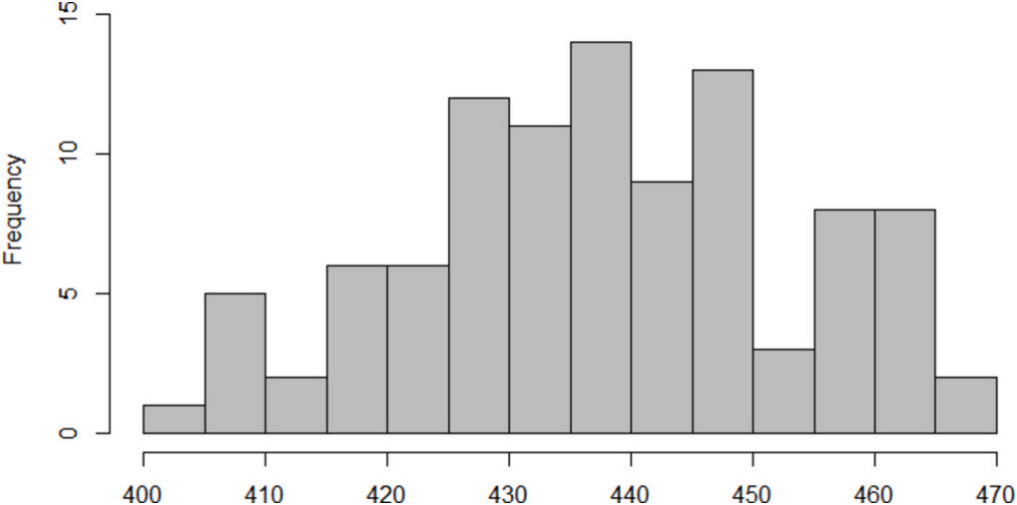
Distribution of the sum of distances between original and jittered positions in each simulation.

Fig. 2 displays the distribution of the distances between the original and shifted locations across the 100 simulated datasets depending of the area type (rural, urban). As expected by the DHS jittering algorithm, the median distance between the true and their jittered locations was larger in rural than urban clusters (See samples in the additional file 2: Fig. A.1, Fig. A.2, Fig. A.3, Fig. A.4, Fig. A.5).

Geostatistical analysis of MIS data

The geostatistical variable selection performed at the cluster level model (Model 1a) fitted on the original MIS data identified NDVI and altitude (in continuous form), EVI and DWB (in categorical form) and the presence of forest (binary) as the most important predictors of parasitaemia risk (Table 1). Estimates of the final geostatistical model (Table 2) indicated that the malaria parasite risk was positively associated with NDVI, EVI, and presence of forest, and negatively associated with altitude. The individual level model (Model 2a) fitted to the original MIS data selected the proportion of households with 1 ITN per 2 persons as the most important predictor (Table 1). The association of this predictor with the parasitaemia risk was negative as shown in the final geostatistical model (Table 2).

**Table 1:** Posterior inclusion probabilities (%) of the climatic predictors and intervention coverage indicators based on the geostatistical variable selection applied to the three datasets i) observed MIS (cluster-level Model 1a, individual-level Model 2a) ii) simulated data with the best predictive ability (Model 1b, Model 2b) and iii) simulated data with worst predictive ability (Model 1c, Model 2c). Inclusion probabilities of the selected predictors are in bold.

Model	Predictor	MIS data			Simulated data with best predictive ability			Simulated data with worst predictive ability		
		Excluded	Continuous	Categorical	Excluded	Continuous	Categorical	Excluded	Continuous	Categorical
Model 1a, 1b, 1c: Cluster level	RFE	36	18	46	56	18	26	26	10	**64**
	NDVI ⁎	12	**83**	5	2	**98**	0	20	**58**	22
	LSTD	55	23	22	59	19	22	60	17	23
	EVI⁎	16	17	**67**	0	0	**100**	12	42	46
	DWB⁎	23	25	**52**	33	26	41	10	14	**76**
	Altitude⁎	1	**96**	3	3	**79**	18	2	**98**	0
	Forest⁎	34	–	**66**	41	0	**59**	36	0	**64**
	Savannah	69	–	31	68	0	32	74	0	26
	Cropland	72	–	28	81	0	19	75	0	25
	LSTN	54	29	17	46	**54**	0	24	10	**66**
Model 2a,2b,2c: Individual level	% of population access to an ITN in their household	84	16	–	87	13	–	82	18	–
	% of households with at least one ITN	100	0	–	84	16	–	98	2	–
	% of households with one ITN per two persons⁎	47	**53**	–	42	**58**	–	77	23	–
	% of children slept under ITN previous night⁎	69	31	–	87	13	–	43	**57**	–
	% of children with fever who received recommended anti-malaria drugs (ACT)	73	27	–	76	24	–	70	30	–

⁎ : the climatic or intervention indicator is selected.

**Table 2:** Estimates (posterior median and 95 % BCI) of the geostatistical model parameters based on the cluster level (Models 1a, 1b, 1c) and the individual level models (Models 2a, 2b, 2c).

Factor		MIS data		Simulated data with the best predictive ability		Simulated data with the worst predictive ability	
		Model 1a	Model 2a	Model 1b	Model 2b	Model 1c	Model 2c
		*OR (95 % BCI)*	*OR (95 % BCI)*	*OR (95 % BCI)*	*OR (95 % BCI)*	*OR (95 % BCI)*	*OR (95 % BCI)*
	0–30 mm					1	1
RFE	30–60 mm					0.83(0.24; 2.49)	2.54(0.86; 7.97)
	>60 mm					0.36(0.08; 1.65)	1.07(0.30; 3.98)
NDVI		1.55 (1.12; 2.12)	1.33(0.97; 1.82)	1.63(1.18; 2.29)	1.31(0.96; 1.82)	1.6 (1.23; 2.09)	1.32(1.03; 1.70)
EVI	<0.21	1	1	1	1		
	0.21–0.38	1.90 (1.03; 3.51)	1.38(0.82; 2.33)	1.95(1.03; 3.67)	1.33(0.79; 2.22)		
	>0.38	1.25 (0.51; 3.02)	0.92(0.41; 2.1)	1.24(0.49; 3.08)	0.9(0.40; 1.98)		
DWB	<70 m	1	1			1	1
	≥ 70 m	1.82 (1.005; 3.45)	1.60(0.90; 2.86)			1.98(1.09; 3.95)	1.74(0.98; 3.17)
Altitude		0.39 (0.26; 0.57)	0.37(0.25; 0.53)	0.53(0.3; 0.91)	0.42(0.25; 0.72)	0.38(0.24; 0.6)	0.35(0.23; 0.54)
Forest	No	1	1	1	1	1	1
	Yes	1.55 (1.002; 2.39)	1.17(0.77; 1.79)	1.49(0.95; 2.34)	1.18(0.77; 1.81)	1.46(0.93; 2.28)	1.06(0.68; 1.62)
LSTN_continuous				1.37(0.83; 2.29)	1.19(0.76; 1.89)		
	0–14					1	1
LSTN_categrical	14–18					1.82(0.29; 18.01)	2.06(0.63; 14.04)
	>18					1.45(0.21; 16.22)	1.87(0.53; 13.42)
Gender	Female		1		1		1
	Male		0.99(0.86; 1.15)		0.99(0.86; 1.15)		1(0.87; 1.15)
Area type	Rural		1		1		1
	Urban		0.55(0.38; 0.80)		0.54(0.38; 0.78)		0.56(0.39; 0.81)
Wealth Index	Most poor		1		1		1
	Very poor		0.60(0.46; 0.76)		0.6(0.47; 0.76)		0.6(0.47; 0.76)
	Poor		0.66(0.49; 0.89)		0.66(0.48; 0.88)		0.65(0.48; 0.87)
	Less poor		0.46(0.32; 0.66)		0.45(0.31; 0.65)		0.46(0.32; 0.66)
	Least poor		0.39(0.25; 0.61)		0.39(0.25; 0.61)		0.38(0.25; 0.60
Education level of mothers	No education		1		1		1
	Primary		1.15(0.92; 1.43)		1.14(0.91; 1.42)		1.15(0.92; 1.44)
	Secondary		0.92(0.70; 1.22)		0.92(0.7; 1.21)		0.93(0.7; 1.22)
	University		1.03(0.57; 1.84)		1.03(0.56; 1.83)		0.98(0.53; 1.73)
Age	0–1+		1		1		1
	1–2		1.31(0.96; 1.77)		1.32(0.97; 1.79)		1.34(0.99; 1.81)
	2–3		2.29(1.70; 3.10)		2.30(1.71; 3.10)		2.32(1.73; 3.12)
	3–4		2.57(1.90; 3.48)		2.59(1.92; 3.49)		2.60(1.93; 3.51)
	>4		3.49(2.62; 4.65)		3.38(2.51; 4.54)		3.40(2.54; 4.57)
% households with 1 ITN per 2 persons			0.16(0.05; 0.47)		0.14(0.05; 0.44)		
% of children with fever in the last two weeks who received ACT							0.35(0.18; 0.66)


Spatial parameters	Posterior median	Posterior median	Posterior median	Posterior median	Posterior median	Posterior median
	(95 % BCI)	(95 % BCI)	(95 % BCI)	(95 % BCI)	(95 % BCI)	(95 % BCI)
Spatial variance	1.81 (1.24; 2.92)	1.62(1.10; 2.76)	1.88(1.22; 3.6)	1.64(1.17; 2.5)	1.87(1.29; 3.13)	1.64(1.1; 2.8)
Range (km) ^1^	154.8 (89.50; 292.96)	188.09(100.35; 353.63)	111.43(62.26; 217.13)	214.98(120.37; 487)	143.29(75.72; 301.36)	158.87(94.18; 321.1)

1: Smallest distance that spatial correlation is <5 %.

### 3.2 Geostatistical analysis of simulated data

Geostatistical variable selection applied to each of the simulated data identified 26 sets of climatic and environmental predictors that were included in the selected model (Table A.2 in Appendix). Two simulated models among one hundred had the highest posterior inclusion probabilities equal to 19 % and 18 %. Both models included NDVI and altitude (continuous), EVI (categorical) and forest presence. Furthermore, the DWB was included in the second most frequent model (with inclusion probability of 18 %).

In accordance with the original (un-jittered MIS data), all simulated data identified the altitude (in continuous form) as an important predictor, while the LSTD, savannah and cropland land use types were excluded from all the selected models (Table 3). LSTN was rarely included in the set of important predictors.

**Table 3:** Relative frequencies of the climatic predictors and their functional forms identified by the geostatistical variable selection across the 100 simulated data. The predictors selected by the original data are in bold.

Climatic predictors										
Functional form	RFE	NDVI	LSTD	EVI	DWB	Altitude	Forest	Savannah	Cropland	LSTN
Continuous	0 %	**95 %**	0 %	24 %	0 %	**100 %**	0 %	0 %	0 %	1 %
Categorical	25 %	1 %	0 %	**51 %**	**64 %**	0 %	**90 %**	0 %	0 %	3 %
Excluded	75 %	4 %	**100 %**	25 %	36 %	0 %	10 %	**100 %**	**100 %**	**96 %**

**Fig. 3:**
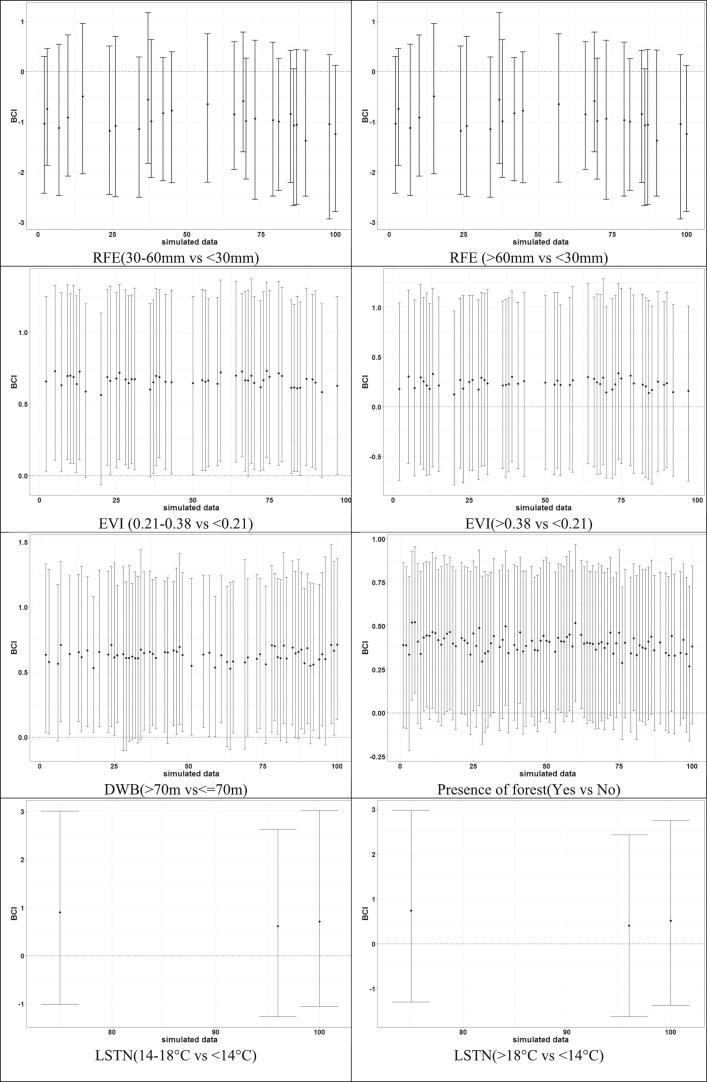
Effects (posterior median, 95 % BCI) of the categorical covariates estimated by the selected geostatistical model for each simulated data (1−100) ordered according to the logarithmic predictive score values and of the data 0 corresponding to the observed data.

**Fig. 4:**
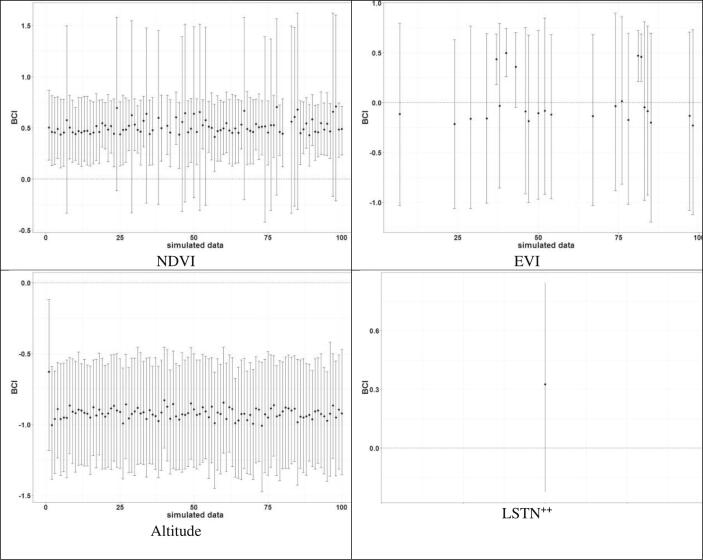
Effects (posterior median, 95 % BCI) of continuous covariates estimated by the selected geostatistical model for each simulated data ordered according to the logarithm predictive score values of the models.

The estimates of the effects of climatic predictors based on the selected models were overlapping between the simulated datasets (Fig. 3, Fig. 4). Altitude (in continuous form) was always statistically important and negatively associated with the parasitaemia risk. NDVI was positively associated and statistically important for the malaria parasite risk in most simulated data. Malaria parasite risk had a positive and most often statistically important relationship with the presence of forest, EVI and DWB. On the other hand, the importance of RFE, LSTN or LSTD on parasitaemia risk varied with the data.

Table 1 presents posterior inclusion probabilities of the selected models based on the simulated data with the best (Model 1b) and worst predictive performance (Model 1c) and on the observed MIS data (Model 1a). The difference between Model 1b and Model 1a was that the former included LSTN and excluded DWB. Model 1c included RFE, LSTN which were not in Model 1a and excluded EVI. Regarding the selection of intervention indicators from the individual-level model, the Model 2b with the best predictive performance among the simulated data gave similar results to the true model (Model 2a). The model with the worse performance among simulated data (Model 2c) was not able to capture the statistically important effect of the malaria intervention indicator (i.e. proportion of households with 1 ITN per 2 persons and the proportion of children who slept under an ITN the previous night). The direction of the effects was estimated by the Bayesian geostatistical models (Table 2).

**Fig. 5:**
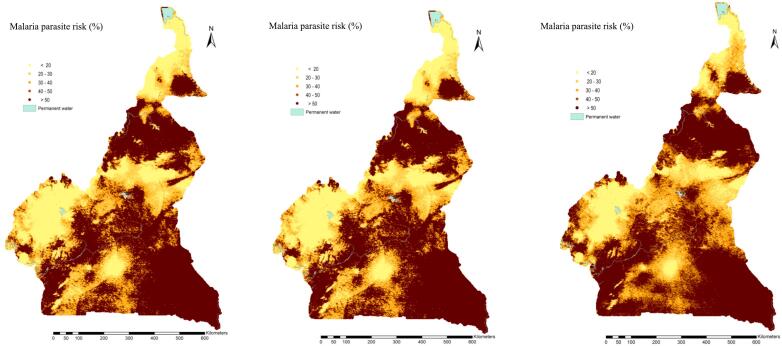
: Malaria parasite risk estimates (median of predictive posterior distribution) among children less than 5 years, obtained from i) Model 1a (left), ii) Model 1b (center) and iii) Model 1c (right).

**Fig. 6:**
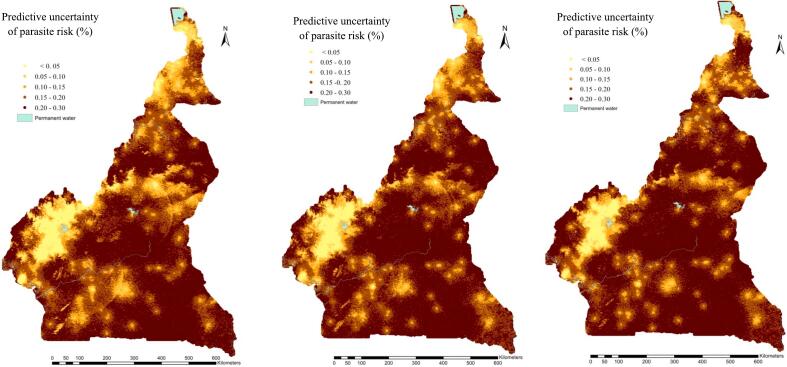
: Predictive uncertainty (standard deviation of predictive posterior distribution) of estimated parasite risk among children less than 5 years, obtained from i) Model 1a (left), ii) Model 1b (center) and iii) Model 1c (right).

The global spatial patterns of disease risk in the East, North and Coastal parts of the country were well captured by the three cluster-level models. Maps drawn on the same scale clearly indicated similar geographic patterns predicted by the three models (Model 1a, Model 1b, and Model 1c), therefore the models with best and worst predictive performance were able to capture the disease risk distribution of the MIS dataset. However, the prediction uncertainties of Model 1b and Model 1c over the gridded surface were greater than the ones obtained from Model 1a (Fig. 5, Fig. 6).

The spatial variance estimates and uncertainty obtained from the simulated data with the worst and best predictive abilities were close to the ones produced by the observed MIS data. The residual spatial correlation estimated by the different clusters and individual models remained high, indicating the presence of unmeasured spatially structured factors related to the geographic distribution of the parasitaemia risk.

## 4 Discussion

This study is the first to assess the effects of jittering of DHS/MIS cluster locations on the estimates of the geographical distribution of malaria risk and of the intervention effects obtained by Bayesian geostatistical modelling (Adigun et al., 2015, Diboulo et al., 2016, Giardina et al., 2014, Gosoniu et al., 2012). A large number of jittered datasets were simulated from real data and geostatistical variable selection was applied to determine the impact of jittering on the model formulation.

Different subsets of climatic factors in the simulated data were identified as important predictors of malaria risk. However, in 18 % of the datasets, the models included the same predictors with the fitted model obtained by the observed MIS data, while the model with the highest posterior inclusion probability (19 %) could not capture the statistically importance of the DWB predictor. NDVI and altitude were selected in more than 95 % of the simulated data and DWB was identified in 64 %. Furthermore, the jittering of cluster locations had an influence on the selected functional form of the climate predictors (continuous/categorical). These results showed that spatial displacement can influence the risk factor analysis and the estimation of the effects of malaria interventions on the disease risk. Similar findings have been reported for distance-based covariates in the study presented by Warren et al. (Warren et al., 2016b).

The direction of the relation between parasite risk, NDVI and altitude remained the same in all simulated data. In particular, the continuous form of NDVI was statistically important in most of simulations and as expected, the altitude was always negatively related and statistically important to the malaria parasite risk. Those associations were confirmed with the estimates obtained from the true dataset and findings from others studies (Adigun et al., 2015, Samadoulougou et al., 2014). The jittering did not affect the direction of the relationship between malaria risk, NDVI and Altitude. However, the jittering had an effect on the uncertainty estimates of the covariate effects and therefore on their statistical importance. Warren et al. have also concluded that displacement of clusters led to an increase in the estimated uncertainty of the regression coefficient (Warren et al., 2016a). In addition, Cressie et al. proved that in the presence of spatial location error, the prediction estimates and regression coefficients were influenced (Cressie and Kornak, 2003).

In most simulations, the BCIs of the altitude and NDVI (continuous forms), EVI and DBW (categorical forms) were overlapping. The majority of datasets were able to capture the statistical importance of those covariates. Altitude and vegetation index change little in the space within the radius corresponding to the random displacement of cluster's coordinates, most likely due to small environmental gradient within each ecological zone of the country. Thus, locations inside the displacement buffer shared in most cases the same environmental conditions and therefore their parameter estimates were not affected by jittering (Kwan, 2012).

The simulated data with the highest predictive performance was the one with location configuration among the closest to the true data. The parameter estimates of this model were also similar to the fitted model on true data.

According to the simulation, the proportion of households with 1 ITN per 2 persons or the proportion of children who slept under an ITN the previous night before the survey were identified as important predictors of the individual-level malaria risk model. Variable selection applied on the intervention coverage indicators revealed that jittering influenced their posterior inclusion probabilities into the model and therefore the inference about the effects of malaria interventions. This result could be due to the confounding effects of climatic predictors.

All the wealth index categories were statistically important and negatively associated to the malaria parasite risk in the true model. The three individual level models showed that, posterior parameter estimates of socioeconomic factors were relatively stable, irrespective of the model. The socioeconomic factors were related to the individual risk rather than the malaria prevalence at the location level, and thus estimates of the socio-economic effects were not much influenced by the displacement of the clusters. Similarly, demographic factors also related to the individual were not affected by the jittering. The gender was not statistically important and a gradient of risk was noted in the age groups as already supported by other studies (Gosoniu et al., 2012; Adigun et al., 2015; Diboulo et al., 2016; Ssempiira et al., 2017).

The effect of the selected intervention indicator was statistically important and negatively associated to the parasitaemia risk regardless of the simulated dataset. Similar to the socioeconomic status and demographic factors, intervention effects were more likely to be higher at the individual and household than the community; therefore the changes of cluster locations did not influence the direction of the relationship between ITN coverage indicators and malaria parasite risk after adjusting for socioeconomic factors.

The BCIs of the spatial correlation parameters of the true, best and worst models were overlapping and their spatial variances were not dramatically changed. Spatial range parameters depend on the cluster locations and were sensitive to the distance between locations. The individual level models overestimated spatial correlation especially for the model having the worst predictive ability. The change of cluster locations could lead to a misspecification of the spatial dependence structure of the disease risk (Warren et al., 2016b; Perez-Haydrich et al., 2013).

The geographical patterns obtained from the simulated data with the highest and lowest predictive performance were similar to the ones obtained from the true data. The relationship between malaria risk and the climatic factors was rather stable within the same ecological zone and therefore was not strongly influenced by the jittering of the locations. This result was expected since several studies showed a local interdependency between climatic factors within small buffer zones. (Berg et al., 2013; Haerter et al., 2024, Hou et al., 2018).

These results were based on the assumptions of a stationary and isotropic spatial process of the malaria risk. Violation of these assumptions may influence the results of geostatistical variable selection and therefore the impact of jittering on model specification.

The major overall limitation of this study was that it focussed on a single dataset from a single country, examining a specific outcome within two quite specific modelling frameworks. However, Cameroon settings encompass most of the African settings which include Sahelian, Semi-Sahelian, Cold and Forest area. The changes observed on malaria risk estimates and on interventions predictors obtained from non-stationary geostatistical models could be potentially generalized in the other African contexts (Chouakeu et al., 2023, Fondjo et al., 2023).

## 5 Conclusion

Moderate spatial modifications in the geographical positions of the clusters surveyed might have little influence on the estimation of the spatial patterns of malaria risk in Cameroon, especially when the climatic and environmental conditions are similar within the radius of the random displacement of locations. Nevertheless, the jittering of cluster locations has an impact on the selection of climatic predictors used to estimate the disease risk at high geographical resolution and could affect the interpretation of the relationship between malaria parasite infection with environmental and climatic factors that support the disease transmission.

## Ethics approval and consent to participate

According to the Cameroon law, the DHS, MICS and MIS are carried out by the National Institute of Statistics, and because of blood samples collection, the clearance of the national ethical committee on health was obtained before the field step of survey. During those surveys, the head of household or the person in charge of children must give their consents before answering of questionnaire and blood screening.

## Funding

This work was supported by the 10.13039/501100000781European Research Council (ERC) IMCCA grant number 323180 and the Swiss National Foundation (SNF) program for Research on Global Issues for Development (R4D) project number IZ01Z0–147286.

## Authors' contribution

PV had conceived, designed the study and contributed to the analysis. KC and RW contributed to the design, collect of the DHS and MIS data. SM had analysed the data and drafted the manuscript. PV, SM, KC and RW revised the manuscript and provided the intellectual content. All authors read and approved the final manuscript.

## CRediT authorship contribution statement

**Salomon G. Massoda Tonye:** Writing – review & editing, Writing – original draft, Visualization, Validation, Software, Resources, Methodology, Investigation, Data curation, Conceptualization. **Romain Wounang:** Methodology, Data curation, Conceptualization. **Celestin Kouambeng:** Writing – review & editing, Validation, Resources, Data curation, Conceptualization. **Penelope Vounatsou:** Writing – review & editing, Supervision, Software, Resources, Project administration, Methodology, Funding acquisition, Data curation, Conceptualization.

## Declaration of competing interest

The authors declare that they have no competing interests.

## Figures and Tables

**Table A.1:** Spatial and temporal resolution of climate and environmental factors.

Data	Source	Spatial resolution (km^2^)	Temporal frequency
Land surface temperature (LST) for day and night^1^	Moderate resolution Imaging Spectroradiometer (MODIS) Terra	1 × 1	8 days
Normalized difference vegetation index (NDVI) 1	MODIS	0.25 × 0.25	16 days
Enhanced vegetation index (EVI)^1^	MODIS	1 × 1	16 days
Rainfall estimates (RFE) 2	U.S. Geological Survey-Earth Resources Observation Systems (USGSS)	8 × 8	10 days
Land cover 1: Cropland, Forest, Savannah	MODIS	1 × 1	year
Digital elevation (Altitude)^3^	Shuttle Radar Topographic Mission(SRTM)	1 × 1	NA
Permanent water bodies (rivers, lakes, wetlands) 1	MODIS	1 × 1	NA

1 . http://reverb.echo.nasa.gov/reverb

2 . http://earlywarning.usgs.gov/fews/

3 . http://glcfapp.glcf.umd.edu/data/srtm/

**Table A.2:** Frequency distribution of the best models identified by the geostatistical variable among the 100 simulated data. Model M1a includes the combination of predictors included in the best model obtained from the observed MIS data.

	Climatic and environmental factors ⁎										
Model	RFE	NDVI	LSTD	EVI	DWB	Altitude	Forest	Savannah	Cropland	LSTN	Frequency
**1**	**1**	**2**	**1**	**3**	**1**	**2**	**3**	**1**	**1**	**1**	**19 %**
**2**	**1**	**2**	**1**	**3**	**3**	**2**	**3**	**1**	**1**	**1**	**18 %**
3	3	2	1	3	3	2	3	1	1	1	9 %
4	1	2	1	1	3	2	3	1	1	1	8 %
5	1	2	1	2	3	2	3	1	1	1	6 %
6	3	2	1	2	3	2	3	1	1	1	5 %
7	1	2	1	2	1	2	3	1	1	1	5 %
8	1	2	1	1	1	2	3	1	1	1	5 %
9	3	2	1	1	3	2	3	1	1	1	4 %
10	1	2	1	1	3	2	1	1	1	1	3 %
11	3	1	1	2	3	2	3	1	1	1	2 %
12	1	2	1	3	3	2	1	1	1	1	2 %
13	3	2	1	3	1	2	3	1	1	1	1 %
14	3	2	1	2	3	2	1	1	1	1	1 %
15	3	2	1	1	3	2	3	1	1	3	1 %
16	3	2	1	1	3	2	1	1	1	1	1 %
17	3	2	1	1	1	2	3	1	1	1	1 %
18	1	3	1	2	3	2	3	1	1	1	1 %
19	1	2	1	3	1	2	3	1	1	3	1 %
20	1	2	1	3	1	2	3	1	1	2	1 %
21	1	2	1	2	3	2	1	1	1	1	1 %
22	1	2	1	2	1	2	1	1	1	1	1 %
23	1	2	1	1	3	2	3	1	1	3	1 %
24	1	2	1	1	1	2	1	1	1	1	1 %
25	1	1	1	2	3	2	3	1	1	1	1 %
26	1	1	1	2	1	2	3	1	1	1	1 %
**M1a**	**1**	**2**	**1**	**3**	**3**	**2**	**3**	**1**	**1**	**1**	

⁎ Legend: 1 = Excluded; 2 = Continuous; 3 = Categorical form.

## Data Availability

MIS were handling technically by the NIS and the NMCP in Cameroon. The MIS dataset is freely available, and any request of information can be sent directly to the NIS or the NMCP.

## Appendix A Additional file 1 Geostatistical models formulation

Different assumptions were made about the probability density functions used at cluster and at individual levels. Models describe below were applied for the true and simulated data, where the index $\mathrm{k\epsilon}\left\{1\ldots 101\right\}$ represents the original data for the first value and the next others the simulated data.

### A.1 Model 1: Estimating malaria risk at cluster level

Considered $Y_{i}^{k}$ the variable of number of parasitaemia positive children among the $n_{\mathrm{ik}}$ screened at the cluster i of the simulation *k,* $s_{i}^{k},\left(i=1,\ldots ,N\right)$*.* In the Bayesian inference context, we had assumed that $Y_{\mathrm{ik}}$ arise from a binomial distribution, i.e. $Y_{i}^{k}∼\mathrm{Bin}\left(n_{i}^{k},p_{i}^{k}\right)$, where the malaria parasite risk at cluster i of the simulation *k,*$\;p_{\mathrm{ik}}$, on the logit scale is a function of climatic factors $X_{i}^{k}=\left(1,X_{i1}^{k},X_{i2}^{k},\ldots X_{\mathrm{ip}}^{k}\right)$ and spatial random effects $U_{\mathrm{ik}}$, such as $\text{logit}\left(p_{\mathrm{ik}}\right)=\sum_{j=0}^{p}\beta_{j}^{k}X_{\mathrm{ij}}^{k}+U_{i}^{k}$.

We assume that $U_{i}^{k}$ arise from a stationary isotropic spatial process i.e. $U^{k}∼\mathrm{MVN}\left(0,\Omega^{k}\right)$, where $U^{k}={\left(U_{1}^{k},U_{2}^{k},\ldots ,U_{N}^{k}\right)}^{T}$ and each $\Omega^{k}\;$is the variance-covariance matrix *N*x*N*, so that $\Omega_{\mathrm{ij}}^{k}={\sigma_{k}}^{2}\;\exp\left(-\rho_{k}d_{\mathrm{ij}}^{k}\right)$. $d_{\mathrm{ij}}^{k}$ is the euclidian distance between cluster $s_{i}^{k}$ and $s_{j}^{k}$, $\sigma_{k}^{2}$ is the spatial variance also called the partial sill and $\rho_{k}\;$the smoothing parameter to control correlation. We consider non-informative prior for $\beta_{j}^{k}$ defined such as $\beta_{j}^{k}∼N\left(0,100\right).$

### A.2 Model 2: Estimating malaria risk at individual level

Let $Y_{\mathrm{ij}}^{k}\;$be the status of malaria parasitaemia of child j who lives in cluster i of the simulation *k,* $s_{i}^{k},\left(i=1,\ldots ,N\right)$*.* We assume a Bernoulli distribution, i.e. $Y_{\mathrm{ij}}^{k}∼\mathrm{Be}\left(\;p_{\mathrm{ij}}^{k}\right)$, and model individual level function covariates on the logit scale of child specific risk,$\;p_{\mathrm{ij}}^{k}$ i.e.$\;\text{logit}\left(p_{\mathrm{ij}}^{k}\right)=\sum_{l=0}^{p}\beta_{j}^{k}X_{\mathrm{ijl}}^{k}+U_{i}^{k}$ where,$\;X_{\mathrm{ij}}^{k}=\left(1,X_{\mathrm{ij}1}^{k},X_{\mathrm{ij}2}^{k},\ldots X_{\mathrm{ijp}}^{k}\right)$,the list of covariates including climatic factors and intervention coverage indicators, $U_{i}^{k}$ is the dependence spatial effect of cluster i of the simulation *k,*$s_{i}^{k}$. Similar prior distributions in Model 1 were also used for spatial and regression coefficients parameters.

### A.3 Prior distributions of the spatial process parameters

We assigned to ${\sigma_{k}}^{2}$ an Inverse Gamma prior distribution,$\;{\sigma_{k}}^{2}∼\mathrm{IG}(2.01,1.01$) and adopt a Uniform prior distribution for$\;\rho_{k}$, i.e. $\rho_{k}∼U\left(\frac{-\log\left(0.05\right)}{{d^{k}}_{\max}},\frac{-\log\left(0.05\right)}{{d^{k}}_{\min}}\right).$The later prior assumes that the spatial correlation bellow 0.05 is negligible, ${d^{k}}_{\max}$ and ${d^{k}}_{\min}$ were corresponding to the maximum and minimum (non-zero) euclidean distance between the survey locations in the same simulations or in the true data.

#### A.3.1 Bayesian variable selection

For each simulated and true data, we have applied stochastic search variable selection assuming a normal mixture prior distribution for the regression coefficient of each climate and intervention factors. A categorical indicator $I_{p}^{k}$ was used to exclude the associated factor *p* from the model of data set *k,* when$\;I_{p}^{k}=0$, to select its continuous form when $I_{p}^{k}=1\;$or to select the categorical form when$\;I_{p}^{k}=2$. We assume a multinomial distribution prior for $I_{p}^{k}\;$with a probability function $\prod_{l=0}^{2}{\pi_{l}^{k}}^{\delta_{l}^{k}\left(I_{p}^{k}\right)}$, where $\pi_{l}^{k}$ represent the inclusion probability of each form (*l* = 0, 1, 2) and $\delta_{l}^{k}\left(.\right)$ is the Dirac function: $\delta_{l}^{k}\left(I_{i}^{k}\right)=\left\{\begin{array}{c} 1\;\mathrm{if}\;I_{i}^{k}=l\;\\ 0\;\mathrm{if}\;I_{i}^{k}\neq l \end{array}\right)$. We adapted a spike and slab prior for the regression coefficients$\;\beta_{p,l}^{k}$, which is a mixture of normal prior distributions i.e. $\beta_{p,l}^{k}∼\delta_{l}^{k}\left(I_{p}^{k}\right)N\left(0,{\tau_{p,l}^{k}}^{2}\right)+\left(1-\delta_{l}^{k}\left(I_{p}^{k}\right)\right)N\left(0,c\times {\tau_{p,l}^{k}}^{2}\right)$ with a mixing proportion equal to the inclusion probability of the corresponding coefficient. The spike component shrinks the regression coefficient to zero when the variable is excluded and the slab assumes a non-informative, normal prior distribution. A climate or intervention factor was identified as important and thus selected in the best model when the inclusion probability of a specific functional form (continuous or categorical) is larger than 50 % (Ishwaran & Rao, 2005; O'Hara & Sillanpää, 2009).

For each simulated and true data, a non-informative prior Dirichlet distribution with hyper-parameter$\;\alpha ={\left(111\right)}^{T}\;$is used, then$\;\;\pi^{k}={\left(\pi_{1}^{k},\pi_{2}^{k},\pi_{3}^{k}\right)}^{T}∼\mathrm{Dirichlet}\left(3,\alpha \right)$, the constant c is fixed at 10^−5^ and inverse Gamma prior distribution was adopted for ${\tau_{p,l}^{k}}^{2}∼\mathrm{IG}\left(0.01,0.01\right)$.

#### A.3.2 Bayesian kriging

Let $S_{0}=\left\{S_{01},S_{02}\ldots ,S_{0m}\right\}$ be the centroids of the gridded surface. Children parasitaemia risks were predicted over the gridded surface from the predictive posterior distribution (Sudipto Banerjee, 2015):

$$P\left(\;{\tilde{Y}}_{0}^{k}|{\tilde{Y}}^{k}\right)\;\alpha \int P\left({\tilde{Y}}_{0}^{k}|{\tilde{Y}}^{k},{\tilde{\beta }}^{k},{\tilde{U}}_{0}^{k},{\tilde{U}}^{k}\right)\times P\left({\tilde{U}}_{0}^{k}|{\tilde{U}}^{k},{\sigma_{k}}^{2},\rho_{k}\right)\times P\left({\tilde{U}}^{k},{\sigma_{k}}^{2},\rho_{k}|{\tilde{Y}}^{k}\right)\times P\left({\tilde{\beta }}^{k}|{\tilde{U}}^{k},{\tilde{Y}}^{k}\right)d{\tilde{\beta }}^{k}d{\tilde{U}}^{k}d{\sigma_{k}}^{2}d\rho_{k}$$

For the dataset *k*, ${\tilde{Y}}_{0}^{k}=Y^{k}\left(S_{0}\right)$,where $Y\left(s_{o}\right)∼\mathrm{Bin}\left(N,p_{s_{o}}^{k}\right)$ is the predicted number of children tested positive in the new location $S_{o}$ and the associated covariates $X_{0}.$Conditional to the spatial process and model parameters, with the link relation between the risk and spatial covariate defined as $\text{logit}\left(p_{s_{o}}^{k}\right)=\sum_{j=0}^{p}\beta_{j}^{k}\;X_{s_{o}j}+{\tilde{U}}_{0}^{k}.$
